# Effects of an Educational Intervention Program on Positional Cranial Deformity in Premature Infants

**DOI:** 10.3390/children11030302

**Published:** 2024-03-04

**Authors:** Alexandra Mosca-Hayler, Daniela López-Schmidt, Romina Curotto-Noce, Jorge Cuevas-Aburto, Jaime Vásquez-Gómez, Samuel Durán-Agüero, Juana Borja González, Ximena Diaz-Martínez, Rafael Zapata-Lamana, María Antonia Parra-Rizo, Igor Cigarroa

**Affiliations:** 1 Escuela de Kinesiología, Facultad de Ciencias, Pontificia Universidad Católica de Valparaíso, Valparaíso 2340000, Chile; alexandra.mosca@pucv.cl (A.M.-H.); daniela.lopez@pucv.cl (D.L.-S.); romina.curotto@pucv.cl (R.C.-N.); 2 Centro de Aprendizaje, Universidad Santo Tomás, Los Ángeles 4440000, Chile; jorgecuevas@santotomas.cl; 3 Centro de Investigación de Estudios Avanzados del Maule (CIEAM), Universidad Católica del Maule, Talca 3460000, Chile; jvasquez@ucm.cl; 4 Escuela de Nutrición y Dietética, Facultad de Ciencias para el Cuidado de la Salud, Universidad San Sebastián, Santiago 8330106, Chile; samuel.duran@uss.cl; 5 Programa de Enfermaría, Universidad del Norte, Barranquilla 080020, Colombia; gjuana@uninorte.edu.co; 6 Grupo de Investigación Calidad de Vida, Universidad del Biobío, Chillán 4300818, Chile; xdiaz@ubiobio.cl; 7 Escuela de Educación, Universidad de Concepción, Los Ángeles 4440000, Chile; rafaelzapata@udec.cl; 8 Faculty of Health Sciences, Valencian International University (VIU), 46002 Valencia, Spain; maria.parrar@umh.es; 9 Department of Health Psychology, Faculty of Social and Health Sciences, Campus of Elche, Miguel Hernandez University (UMH), 03202 Elche, Spain; 10 Escuela de Kinesiología, Facultad de Ciencias de la Salud, Universidad Católica Silva Henríquez, La Florida 8240000, Chile; 11 Facultad de Ciencias de la Salud, Universidad Arturo Prat, Victoria 4720000, Chile

**Keywords:** skull, abnormality, cephalometry, plagiocephaly, prematurity, intervention

## Abstract

Positional cranial deformities are associated with prematurity evolving during the first 2 years of life due to the malleable characteristics of the skull, the first year being the main/primary therapeutic window for intervention. The objectives were (a) to describe health characteristics, peri- and postnatal pathologies, and positional cranial deformities in infants enrolled in an early intervention program and (b) to analyze the effects of a parent education-based intervention program on positional cranial deformity in premature infants. A quantitative, analytical, longitudinal study was conducted. It included 103 premature infants enrolled in an early intervention program (EIP) during the year 2017, all under 4 months of corrected age, to whom a parent education-based intervention program was applied. Cranial circumference, cranial width, diagonals, and anteroposterior diameter were measured, and the cranial asymmetry index (CAI) and cephalic index (CI) were calculated at baseline and during two subsequent evaluations separated by a 3-month period. The main results showed that 75.7% of the infants belonged to a very premature gestational age category, and 57.3% had an adequate weight for gestational age. The most frequent pathologies were premature jaundice, premature anemia, and hyaline membrane disease. The most frequent positional cranial deformity was plagiocephaly. The parent education-based intervention program resulted in (1) a significant decrease in the CAI and a significant increase in the IC, (2) plagiocephalies: an increase in the mild category and a decrease in the moderate + severe categories, (3) brachycephalies: a decrease in the absence category and an increase in the moderate + severe category, and (4) dolichocephalies: an increase in the absence category and a decrease in the mild category. In conclusion, the recommended first line of intervention was not enough for this population, and future studies should support the development of national clinical guidelines, where education is complemented with other therapeutic measures.

## 1. Introduction

Positional cranial deformities constitute a frequent pediatric consultation within the first year of life [1,2,3]. These deformities result in a distortion of the cranial shape due to different external pressures, which can develop during gestation or in the early postpartum months [1,4,5,6,7]. Among the most cited risk factors are prematurity, limited mobility of the child during the early months, extended periods of hospitalization, multiple pregnancies, congenital torticollis, and instrumentalized delivery [1,4,5,6,7]. Deformities are classified into different types based on the area where the alteration occurs, with three main groups identified: (a) plagiocephaly, which is the most common type of cranial deformities, and its main characteristic is flattening in the parieto-occipital area; (b) brachycephaly, presenting bilateral occipital flattening that is symmetrical without a side preference; and finally (c) dolichocephaly, involving flattening of the temporo-parietal region [3,4,5]. Positional cranial deformations commonly occur in premature children given the malleable nature of their cranial bones, a proportionally larger cranial mass, and less head and neck motor control, making them more susceptible to cranial molding [1,2,3], with a higher likelihood of occurrence with increasing degrees of prematurity [8]. The World Health Organization (WHO) considers a live birth >37 gestational weeks as premature and categorizes the following: extremely premature if born before 28 weeks of gestation, very premature if born between 28 and 32 weeks of gestational age, and moderate-to-late premature if born between 32 and 37 weeks of gestational age [9].

Positional cranial deformity may be accompanied by other anatomical alterations, such as facial asymmetry, asymmetries in the forehead shape, and changes in the position of earlobes, the jaw, and cheeks [4,8]. Severe cases could exhibit a visual development deficiency, such as astigmatism and strabismus due to delayed visual field development, as well as auditory impairments [4,10]. Additionally, other studies suggest that children with positional cranial deformities may experience delays in gross motor development [9], language, learning difficulties, and attention deficits [11]. However, evidence suggests that there may not be a direct relationship between the presence of positional cranial deformities and compromises in children’s psychomotor development [12,13]. Regarding the evolution of cranial deformities, evidence suggests that they may positively evolve until the age of two, given the subsequent ossification of the cranial mass, making this period a therapeutic window for timely intervention [12].

The medical diagnosis of positional cranial deformities is mainly clinical and can be supported by cost-effective and easily accessible tools such as anthropometric calipers and a digital analysis of photographs [3,7,8,14,15]. Regarding the management of positional cranial deformities, the treatment is contradictory. Most authors agree on the need for early diagnosis within the first trimester of life, which allows for the informing and educating of parents and/or caregivers about guidelines related to head positioning and basic postural management during various activities at home. This is considered the first-line therapy [3,13,16]. The goal of these measures is to reduce the severity of the deformity and prevent progression and possible associated complications [17]. On the other hand, evidence-based guidelines indicate that a combined approach involving positioning measures, physiotherapy for mild and moderate cases, and the use of cranial orthoses for severe cases would be more effective [18].

Currently, there is no consensus on the best indications for the management of positional cranial deformities. Internationally, it has been described that these positional cranial deformities, specifically plagiocephaly, occur in around 15% of children, although this percentage can increase to 38% in extremely preterm patients [1]. Other authors estimate that, being an underdiagnosed condition, it could affect up to 46–48% of infants under the first year of life [5,6]. In Chile, recent data indicate that 96.1% of premature children enrolled in an early intervention program presented positional cranial deformities [19]. However, there is no recorded data in the Chilean population about the follow-up and progression over time, especially in high-risk populations such as preterm infants, whose survival rates have increased in Chile and worldwide [9,15]. In this context, it is of the utmost importance to analyze the effects of the first-line treatment for these positional cranial deformities, as well as to determine whether these measures are sufficient as a therapeutic approach or if additional interventions and greater preventive measures are necessary, especially in high-risk populations. Regarding existing evidence, this study could potentially be the first in Latin America to provide information about the effects of an early intervention program based on parent education on positional cranial deformity in preterm boys and girls.

Given the considerations, the objectives of the present research were (a) to describe health characteristics, peri- and postnatal pathologies, and positional cranial deformities in infants enrolled in an early intervention program (EIP) and (b) to determine the effects of a parent education-based intervention program on positional cranial deformity in premature infants.

## 2. Materials and Methods

### 2.1. Study Design, Population and Sample

This research used a quantitative, non-experimental, analytical, longitudinal design with convenience sampling. The study included boys and girls referred from the Neonatology Follow-up Polyclinic of Dr. Gustavo Fricke Hospital in Viña del Mar city to the EIP affiliated with the School of Physiotherapy at the Pontifical Catholic University of Valparaíso of Chile. The participants were under post-hospital discharge monitoring as part of the Chilean follow-up program for preterm and high-risk infants. In the year 2017, 130 children were referred to the EIP, which aims to prevent, evaluate, diagnose, and treat potential deficits or delays in the development of infants with a history of high risk. Follow-ups and outpatient physiotherapy treatment were conducted during their first three years of life. The children enrolled in the EIP were mainly premature and had neurobiological risk factors such as genetic syndromes and perinatal hypoxia, among others.

During their stay in the neonatology unit at hospital, frequent repositioning was implemented as a preventive measure against the development of positional cranial deformities. The infants included in the research met the criteria of prematurity, underwent anthropometric cranial evaluation upon program entry using a caliper, and had a corrected age of three months and 29 days or less at the time of the initial assessment (measurement 1), resulting in a sample of 103 infants. To assess the progression of cranial deformity, the children underwent two additional head anthropometry evaluations, each separated by a three-month period (measurements 2 and 3). In the follow-up measurements at 3 and 6 months, the sample size remained at n = 79 and n = 63 subjects, respectively. The reduction in the number of participants at each evaluation was due to subjects’ non-attendance on the specified measurement dates or the inability to contact parents and/or primary caregivers.

The study received approval from the Bioethics Committee of Pontifical Catholic University of Valparaíso (protocol code: BIOEPUCV-H150-date of approval: 11 August 2017), and parents and/or caregivers of the children provided informed consent before participating in the research.

### 2.2. Outcomes and Instruments:

#### 2.2.1. Health Outcomes at Birth and Measurement Age

This included gestational age, birth weight, and weight for gestational age according to the intrauterine growth curves widely used in national and international research [20].

Hospitalization duration: this refers to the length of hospital stay, where prolonged hospitalization was greater than 10 days, using the cutoff point established by Gold et al. (2016) as a reference [21]. This corresponds to the corrected age, measured in days, at the time of the first anthropometric evaluations (measurement 1). 

#### 2.2.2. Cranial Anthropometric Measurements

The most common cranial anthropometric measurements were included: cranial circumference (in cm), width, length, major diagonal, and minor diagonal of the cranial (in mm) [17].

#### 2.2.3. Cephalometric Index (CI)

This index allows for the numerical assessment of dolichocephaly or brachycephaly based on Bosch and Costa in 2017 [17]. It is calculated by multiplying by 100 the result of the quotient between the maximum biparietal distance and the antero-posterior distance taken in the midline. Moreover, 80 has been determined as the ideal proportion value. Brachycephaly is classified as mild (CI = 86–90), moderate (CI = 91–100), severe (CI > 100). Dolichocephaly is classified as mild (CI = 70–74), moderate (CI = 60–69), severe (CI < 60).

#### 2.2.4. Cranial Asymmetry Index (CAI)

This evaluates the presence and degree of plagiocephaly using the criteria published by Bosch and Costa in 2017. It is calculated through the difference between the major diagonal and the minor diagonal. A CAI with a higher score indicates greater severity of the deformity [17]. Plagiocephaly is considered mild: 0–10 mm; moderate: 10–20 mm; and severe: >20 mm.

#### 2.2.5. Presence of Peri- and Postnatal Pathologies

This included prematurity jaundice, prematurity anemia, hyaline membrane disease, sepsis, hypocalcemia, intrauterine growth restriction, bronchopulmonary dysplasia, intracranial hemorrhage grade I and II, patent ductus arteriosus, periventricular leukomalacia, hypoxia, other metabolic disorders, intracranial hemorrhage grade III and IV, chromosomal aberrations, and torticollis.

#### 2.2.6. Early Intervention Program (EIP) Based on Parent Education

The instructions followed the preventive and treatment recommendations published by Bosch in 2017, categorized as the primary therapeutic approach on positional cranial deformity [17]. The program should focus on the prevention of positional cranial deformity. The program includes recommendations for the infant’s sleep and wakefulness stage. Furthermore, the program must begin at one month of age, and its application continues according to the milestones of the infant’s psychomotor development. All parents and/or caregivers received (1) a brief educational talk at the time of measurement 1 (approximately 10 min); (2) guidelines for home care (photos and text) regarding head positioning during sleep, daily repositioning within the crib, avoiding sustained head positions during the day, and proper positioning during the use of equipment, with encouragement to alternate support on the occipital area between right and left and to position the baby in a prone position while awake and under the supervision of the parent or primary caregiver; (3) specific instructions (if applicable) for cases of positional cranial deformity observed at measurement 1, such as head rotation to the opposite side, neck stretches, preferential stimulation from the opposite side of the deformity, increased time in prone and lateral decubitus positions, among others; (4) children with psychomotor development delay received physiotherapy as necessary, followed by continued follow-up evaluations; and (5) reinforcement and updating of instructions during measurements 2 and 3. The education session lasted approximately one hour and was carried out by health professionals with experience in the care and treatment of premature infants.

### 2.3. Statistical Analysis Plan

All analyzes were conducted with IBM^®^ SPSS Statistics^®^ version 27 for Windows, New York, NY, USA. Descriptive statistics for qualitative variables were presented using absolute frequency and percentage, while quantitative variables were described using the mean and standard deviation. The Kolmogorov–Smirnov test was employed to assess the normal distribution of the variables analyzed. To determine differences in anthropometric measurements and cranial asymmetry among various assessments, a one-way repeated measures ANOVA was performed. Subsequently, when differences were identified among assessments, estimated marginal means analysis with the Bonferroni test was conducted. A significance level of α = 0.05 was considered, and differences were deemed statistically significant for a *p*-value < 0.05.

## 3. Results

### 3.1. Description of Health, Peri- and Postnatal Pathologies

Table 1 presents indicators of birth health and the presence of peri/postnatal pathologies in the participating infants. The majority were boys (58.3%) with a gestational age (M: 29.9; SD: 2.5 years). Most belonged to a very preterm gestational age category (75.7%), with a birth weight (M: 1403; SD: 446.7 g), and the majority had an adequate weight for gestational age (57.3%). Additionally, it was evident that infants mostly presented with premature jaundice (93.2%), followed by premature anemia (60.2%) and hyaline membrane disease (60.2%).

### 3.2. Effects of an Early Intervention Program Based on Parental Education on Sample

In Table 2, anthropometric measurements and indicators of cranial asymmetry are presented for assessments 1, 2, and 3. A significant decrease in the cranial asymmetry index (CAI) (M: 5.4; SD: 4.8 vs. M: 4.0; SD: 3.6 mm) and a significant increase in the cranial index (CI) (M: 81.2; SD: 4.8 vs. M: 86.4; SD: 6.4 mm) were observed between assessment 1 and 3. Additionally, as anticipated, a significant increase between assessment 1 and assessment 3 was noted in cranial circumference (M: 37.5; SD: 1.9 vs. M: 43.9; SD: 1.6 cm), cranial length (M: 127.3; SD: 6.4 vs. M: 146.1; SD: 6.7 mm), cranial width (M: 103.2; SD: 6.1 vs. M: 126.0; SD: 6.7 mm), major diagonal (M: 126.0; SD: 6.2 vs. M: 144.3; SD: 5.5 mm), and minor diagonal (M: 120.6; SD: 6.3 vs. M: 140.3; SD: 6.1 mm).

In Table 3, the frequency of cranial asymmetries and their severity in measures 1, 2, and 3 are shown. Plagiocephaly was observed as the most common positional cranial asymmetry in infants. For plagiocephalies, an increase was noted in the mild category (Measure 1: 76.7% to Measure 3: 88.9%), while the moderate + severe category decreased (Measure 1: 17.5% to Measure 3: 6.3%). In brachycephalies, the absence category decreased (Measure 1: 81.6% to Measure 3: 52.4%), leading to a consequent increase in the moderate + severe category (Measure 1: 1.9% to Measure 3: 30.2%). For dolichocephalies, the absence category increased (Measure 1: 87.4% to Measure 3: 98.4%), resulting in a decrease in the mild category (Measure: 10.7% to Measure 3: 1.6%).

## 4. Discussion

### 4.1. Main Results of the Study

The study reveals that infants were mainly classified as very preterm (75.7%) with an appropriate weight for gestational age (57.3%). Additionally, infants predominantly showed prematurity-related conditions, with jaundice (93.2%), followed by premature anemia (60.2%), and hyaline membrane disease (60.2%) being the most prevalent. Plagiocephaly emerged as the most common positional cranial asymmetry in infants.

Concerning the effects of the parent-based intervention program, significant reductions in the cranial asymmetry index (CAI) and a noteworthy increase in the cranial index (CI) were observed. Upon analyzing positional cranial asymmetries, a rise in the mild category and a decrease in the moderate + severe categories were noted in plagiocephalies. In brachycephalies, there was a decline in the absence category and a concurrent increase in the moderate + severe category. Lastly, dolichocephalies exhibited an increase in the absence category, leading to a decrease in the mild category.

### 4.2. Health Characteristics, Peri/Postnatal Pathologies, and Frequency of Positional Cranial Deformities

The infant participants in the study exhibited primarily prematurity-related health characteristics such as prematurity-related jaundice, prematurity-related anemia, hyaline membrane disease, and sepsis during the peri- and postnatal periods. Additionally, positional cranial deformities were prevalent, with 94.1% presenting with plagiocephaly, 18.4% with brachycephaly, and 12.6% with dolichocephaly in the initial assessment. These proportions agree with the existing literature, which indicates that plagiocephaly is the most common positional cranial abnormality in infants, followed by brachycephaly and dolichocephaly [22].

### 4.3. Effects of an Early Intervention Program Based on Parental Education on Positional Cranial Deformity

In our study, infants with positional plagiocephaly demonstrated favorable progression, showing a decrease in the cranial asymmetry index (between measurements 1 and 3), despite not moving to a different severity category. Furthermore, there was a reduction in moderate +severe cases and an increase in mild cases. These findings are consistent with the literature, which indicates that educational measures on positioning are effective in promoting cranial remodeling in mild positional plagiocephaly in infants under 4 months of age. However, the infants included in this study did not reach normal ranges in the cranial asymmetry index, suggesting that while these measures are beneficial, they may not be enough to reverse this condition. This may be explained by the high neurobiological risk of the study population as children with high neurobiological risk have been described as having greater difficulty in correcting skull shape in the early years of life [23].

Regarding the cephalic index, an increase was observed between measurements 1 and 3, indicating a trend towards the worsening of mild brachycephalic cranial deformity. Additionally, there was an increase in moderate to severe cases and a decrease in the number of children without brachycephaly. This could be attributed to the positioning measures adopted at home by caregivers. Although there was a particular emphasis on encouraging alternating support, whether in lateral, supine, and prone positions, it is possible that this recommendation was not fully followed by the primary caregivers of these children. This may have involved favoring the symmetric supine positioning and not adhering to the recommended hours in the prone position, indicated to place the child on their stomach several times during the day to reduce constant pressure on the posterior and lateral areas of the skull and stimulate their development. Additionally, the fact that the study group consisted of premature infants, who are at higher risk of reduced spontaneous mobility, may have increased the risk of pressure on the posterior aspect of the skull, providing a possible explanation for these results.

On the other hand, regarding dolichocephalic cranial deformity, a decrease in cases was observed at the end of the follow-up, particularly a reduction in mild cases, with a trend toward normality in the cephalometric index. This aligns with the outcomes observed in plagiocephaly cases and corresponds to the literature, indicating that positioning measures would be beneficial as a first-line treatment in mild cases [23,24].

It is important to remember that this study followed cranial deformity until before 12 months of corrected age. This choice was based on research indicating that the most significant correction of this alteration would occur before 12 months of age, a stage where there is greater skull malleability due to increased brain growth [1]. Beyond one year, correction would continue, although at a slower rate, until 2 years of age [6]. Consequently, it is not possible to determine whether the children enrolled in this EIP will maintain the positional cranial deformity category obtained in measurement 3 or if they will evolve positively or negatively over time.

Given that most children in this study did not achieve total correction of positional cranial deformity, it raises the question of whether educational intervention on positioning measures is sufficient as a primary treatment in this population, especially considering that they are premature infants with high neurobiological risk. While the literature describes educational measures as the treatment for mild cases, some studies suggest that in cases where children have mobility disorders or the cranial deformity is severe, educational measures should be complemented with other therapeutic approaches such as physiotherapy or a referral to a neurosurgery department/pediatric neurosurgeon to evaluate the need for cranial orthoses or surgical treatment [12,18,24,25].

According to existing evidence, there are no studies in Latin America on therapeutic interventions or the evolution of positional cranial deformities in premature infants. Therefore, this study is the first article presenting results on the effects of therapeutic interventions in positional cranial deformities, providing the most updated information on the evolution of these deformities in premature infants participating in an EIP. These results underscore the need for further studies describing the evolution of deformities in this population. Additionally, it encourages healthcare professionals working with premature infants with positional cranial deformities to question if the current treatment is enough or if alternative interventions, such as physiotherapy or the use of cranial orthoses, could be utilized to promote cranial remodeling in these children [24,25,26]. Now, there is extensive scientific evidence that supports physiotherapy and cranial helmet therapy in infant cranial deformities [26,27]. This is particularly important in the context of the public healthcare system in Chile, which currently does not guarantee access to these types of therapies, leaving educational interventions as the therapeutic option for all infants with positional cranial deformities.

### 4.4. Limitations of the Study and Future Lines of Research

Among the main limitations is that the population is confined to a single healthcare center, and the sample size is small, representing only what happens in the EIP at the Pontifical Catholic University of Valparaíso, Chile. It is recommended to replicate this study in other early intervention programs in different cities to establish the prevalence and evolution at the provincial, regional, and national levels of positional cranial deformities, thus giving a better understanding on this issue in public health. The authors acknowledge that it was not possible to associate other risk factors in the analysis, such as the presence of pathologies like prematurity-related jaundice, anemia, hyaline membrane disease, and sepsis. It would be relevant for future studies to focus on determining if there is an association between peri- and postnatal pathologies and the evolution of positional cranial asymmetries in all the babies in the country.

### 4.5. Contributions and Clinical Implications

Regarding existing evidence, this study is the first in Latin America to provide information about the effects of an early intervention program that teaches parents on positional cranial deformity in preterm boys and girls.

Based on the results obtained in this study, it seems that educational intervention for parents regarding positional cranial deformity may not be enough as the sole modality of treatment to address positional cranial deformities in premature infants enrolled in an EIP. This study could encourage health professionals working in early intervention programs to generate new strategies for the care of premature babies with positional cranial deformities. Since positional cranial deformities could be a risk factor for delayed development, interventions such as physiotherapy and orthopedic treatment could help prevent developmental delays in infants [26,27,28].

## 5. Conclusions

Most premature patients enrolled in EIP exhibit positional cranial deformities, particularly plagiocephaly of mild severity. Although the intervention does not achieve a normal range, there is a reduction in the cranial asymmetry index. Furthermore, an increase in brachycephaly cases is observed over time, despite the implemented educational measures. Therefore, it seems that the currently recommended first-line intervention for these cases may not be enough for this population. Future studies should support the development of national clinical guidelines, where education is complemented with other therapeutic measures.

## Figures and Tables

**Table 1:** Indicators of birth health and peri/postnatal pathologies of the sample.

Variables	Total (n = 103)
Sex (%)	
*Girls*	43 (41.7)
*Boys*	60 (58.3)
Gestational age (weeks)	29.9 ± 2.5
Types of birth (%)	
*Cesarean birth*	75 (72.8)
*Vaginal delivery*	25 (24.3)
*No data available*	3 (2.9)
*Assisted vaginal delivery (vacuum or forceps)*	0 (0)
Gestational age category (%)	
*Moderate to late preterm*	8 (7.8)
*Very preterm*	78 (75.7)
*Extremely preterm*	17 (16.5)
Birth weight (grams)	1403.0 ± 446.7
Birth weight category (%)	
*Adequate for gestational age*	59 (57.3)
*Small for gestational age*	16 (15.5)
*Severe small for gestational age*	20 (19.4)
*Large for gestational age*	8 (7.8)
Hospitalization time (days)	56.1 ± 29.9
Age at first assessment (days)	38.4 ± 24.9
Peri and postnatal pathologies (%)	
Premature jaundice	96 (93.2)
Premature anemia	60 (60.2)
Hyaline membrane disease	60 (60.2)
Sepsis	49 (47.6)
Hypocalcemia	34 (33.0)
Intrauterine growth restriction	31 (30.1)
Bronchopulmonary dysplasia	23 (22.3)
Intracranial hemorrhage grade I and II	18 (17.5)
Persistent ductus arteriosus	11 (10.7)
Periventricular leukomalacia	9 (8.7)
Hypoxia	6 (5.8)
Metabolic disorders	7 (6.8)
Intracranial hemorrhage grade III and IV	4 (3.9)
Chromosomal disorders	0 (0)
Congenital torticollis	0 (0)

Note: Qualitative variables are presented in frequency and percentage (%) and quantitative variables in mean ± standard deviation. n = 103.

**Table 2:** Anthropometric measures and cranial asymmetry indicators at assessments 1, 2, and 3.

	Measure 1	Measure 2	Measure 3	One-Way Repeated Measures ANOVA	
	M (SD)	M (SD)	M (SD)	ES	*p*-Value
Anthropometric variables					
Cranial Circumference (cm)	37.5 (1.9) a	43.4 (12.7) b	43.9 (1.6) b	0.193	<0.0001
Cranial Length (mm)	127.3 (6.4) a	138.9 (6.2) b	146.1 (6.7) c	0.884	<0.0001
Cranial Width (mm)	103.2 (6.1) a	119.2 (6.7) b	126.0 (6.7) c	0.926	<0.0001
Major Cranial Diagonal (mm)	126.0 (6.2) a	137.6 (5.2) b	144.3 (5.5) c	0.878	<0.0001
Minor Cranial Diagonal (mm)	120.6 (6.3) a	132.5 (5.7) b	140.3 (6.1) c	0.873	<0.0001
Asymmetry variables					
Cranial Asymmetry Index (CAI)	5.4 (4.8) a	4.9 (4.0) a	4.0 (3.6) a	0.057	0.034
Cranial Index (CI)	81.2 (4.8) a	85.9 (6.0) b	86.4 (6.4) b	0.496	<0.0001

Note: SD: standard deviation, ES: effect size. The analysis was conducted using a one-way re-peated measures ANOVA. Mean values within a row with a different letter (a–c) indicate sig-nificant differences between groups (comparison with Bonferroni test). A *p*-value of less than 0.05 was considered statistically significant for all analyses.

**Table 3:** Cranial asymmetry—plagiocephaly, brachycephaly, and dolichocephaly in three measurements.

	Measure 1 (n = 103)	Measure 2 (n = 79)	Measure 3 (n = 63)
Cranial Asymmetry Severity Categories	n (%)	n (%)	n (%)
Plagiocephaly			
*Absence*	6 (5.8)	3 (3.8)	3 (4.8)
*Mild*	79 (76.7)	65 (82.3)	56 (88.9)
*Moderate + Severe*	18 (17.5)	11 (13.9)	4 (6.3)
Brachycephaly			
*Absence*	84 (81.6)	39 (49.4)	33 (52.4)
*Mild*	17 (16.5)	23 (29.1)	11 (17.5)
*Moderate + Severe*	2 (1.9)	17 (21.5)	19 (30.2)
Dolichocephaly			
*Absence*	90 (87.4)	79 (100)	62 (98.4)
*Mild*	11 (10.7)	0 (0)	1 (1.6)
*Moderate + Severe*	2 (1.9)	0 (0)	0 (0)

Note: Qualitative variables are presented in frequency and percentage (%). n = 103.

## Data Availability

Data available on request due to restrictions privacy. The data presented in this study are available on request from the corresponding author. The data are not publicly available due to the inclusion of sensitive health information of the neonates who participated in the study.
